# The Story of the Insane from Year to Year

**Published:** 1894-10-27

**Authors:** 


### THE STORY OF THE INSANE FROM YEAR TO YEAR.

(7th Series.)

### GLAMORGAN COUNTY ASYLUM.

The number of patients increased considerably during the year, and on December 31st it stood at 1,089. There were 287 admissions, 77 recoveries, and 94 deaths. The recoveries were 27·1 per cent. on the admissions, and the deaths 7·2 per cent. on the average number resident. From Table X. we learn that hereditary influence accounts for 91 of the year's admissions and drink for 81, so that these two causes are responsible for more than half the insanity of Glamorganshire, and unfortunately Glamorganshire is no exception. We would ask in all seriousness how much longer people with the insane taint are to be allowed to marry, and how much longer people are to go on drinking themselves into madness?

Cerebral and spinal diseases account for 40 of the deaths, and consumption for 19. The admissions included 22 idiots, and Dr. Pringle does not approve of this imbecile element in his asylum, thinking, truly, that it has a bad effect on the other patients. He suggests the union of several counties for the purpose of building accommodation for this class of the insane, but he will find the preliminaries of this step drag themselves out to such a length that the question would not be solved in his lifetime. Why not build a block for idiots, as has been done at Berry Wood, Fareham, and Tooting? The committee express the "sense of their high approval of the manner in which the multiplex duties in connection with both asylums have been carried out by their eminent medical superintendent." The weekly cost of maintenance is 8s. 3½d. per head.

### DERBY BOROUGH ASYLUM AT ROWDITCH.

On January 1st, 1893, this asylum contained exactly 300 patients, and by December 31st the number had risen to 306. There were 94 admissions, 48 recoveries, and 34 deaths. The recoveries are 57·1 per cent. of the admissions, and the deaths are 11 per cent. of the average number resident. The recovery rate is very high indeed, and it is not exceptional, for we notice that it was 51·1 in 1892, and 49·1 in 1891, while the average since the opening of the asylum is 47·2. Twenty-six of the admissions owe their insanity to hereditary influence and 18 to drink. On looking over Table V. we see that 18 of the deaths were from cerebral and spinal diseases, and 5 from consumption. Of the 306 patients, not fewer than 94 are from districts beyond the borough, and as each of these patients is paid for at the rate of 14s. a week, and the actual cost of maintenance is under 10s., it will be seen that Dr. Macphail is making hay while the sun shines, and that Stafford and Denbigh, whence most of their foreign patients come, are suffering to a corresponding amount for their dilatoriness in providing sufficient room for their patients. In asylums as in other things it is the early bird that catches the worm. Dr. Macphail has continued his instructions to the staff, and he reaps the benefit of the trouble involved in lecturing and teaching; for we find him saying the attendants and nurses are more helpful to the medical officers in the treatment of individual cases. Eight members of the staff have received the certificate of the Medico-Psychological Association, but only after two years' training, so that as regards duration of training they are not on a level with the best hospitals. The committee mention "their satisfaction with the management and discipline of the institution under the care and direction of Dr. Macphail." The weekly rate of maintenance is 9s. 11d. per head.

### THE EARLSWOOD ASYLUM FOR IDIOTS.

At the beginning of the year this asylum contained 589 inmates, and at the close of the year the numbers had fallen to 581. There were 62 admissions, 44 were discharged relieved, and 26 died. The causes of the idiocy or imbecility are in Table II. divided into parental—and under this head we find that 12 are put down to heredity and consanguineous marriage; into maternal—and under this head we notice three caused by difficult labour, and 23 by shocks or fright received during pregnancy; into infantile—and under this head we find convulsions, accident, fevers, premature birth, &c. Seven of the deaths were caused by pneumonia, and two by consumption. An epidemic of scarlet fever appeared, and 96 of the patients and staff were attacked. The disease seems to have been of a mild type, and there was only one death from it. "The board regret that so few visitors, comparatively, inspect the asylum. The schools, workshops, occupations, and methods employed are deeply interesting, and most of those who have been over the institution are impressed with the beneficent work carried on." We gladly give publicity to the above paragraph, and we hope that all those interested in such questions will take the trouble of going over Earlswood, which was the first asylum of its kind in England, if not in Europe. The committee also "bear testimony to the skill and energy of the medical superintendent and the staff generally."

